# Risk Factors of Residual Dizziness After Successful Treatment for Benign Paroxysmal Positional Vertigo in Middle-Aged and Older Adults

**DOI:** 10.3389/fneur.2022.850088

**Published:** 2022-09-13

**Authors:** Wei Fu, Feng He, Ya Bai, Xinyue An, Ying Shi, Junliang Han, Xiaoming Wang

**Affiliations:** ^1^Department of Geriatrics, Xijing Hospital, Air Force Medical University, Xi'an, China; ^2^Department of Neurology, Xijing Hospital, Air Force Medical University, Xi'an, China

**Keywords:** benign paroxysmal positional vertigo, residual symptoms, clinical characteristics, risk factors, elderly

## Abstract

**Objective:**

The purpose of this study was to analyze risk factors of residual dizziness (RD) after successful treatment for benign paroxysmal positional vertigo (BPPV) in middle-aged and older adults.

**Methods:**

181 patients with BPPV, after successful canalith repositioning maneuver (CRM) treatment, were recruited. All patients were divided into the middle-aged group (aged 45–59 years, *n* = 101) and the older group (over 60 years, *n* = 80). The clinical characteristics were recorded, including age, gender, numbers of maneuvers, involved canal, affected side, RD, comorbidities, dizziness handicap inventory score, and generalized anxiety disorder's 7-item scale score.

**Results:**

The incidence of RD in the older group was significantly higher than that of the middle-aged group (*p* = 0.033). Multivariate logistic regression analysis shows that age (odds ratio = 1.042, *p* = 0.006), moderate to severe dizziness (odds ratio = 2.017, *p* = 0.034), and moderate to severe anxiety (odds ratio = 2.228, *p* = 0.017) were independently associated with RD in middle-aged and older adults.

**Conclusion:**

Older adults exhibited higher incidence of RD after successful treatment for BPPV. Age, moderate to severe dizziness, and moderate to severe anxiety were independent risk factors of RD in middle-aged and older adults.

## Introduction

Benign paroxysmal positional vertigo (BPPV) is the common cause of peripheral vertigo. An epidemiological study revealed that the lifetime prevalence was 3.2% in females, 1.6% in males, and 2.4% overall, with an incidence of 0.6% per year (1). The prevalence of BPPV increases with age and patients older than 60 years of age are almost seven times higher in comparison with those 40-year-old patients (1). Treatment of BPPV relies on canalith repositioning maneuver (CRM). Despite the high success rate of the CRM in treating BPPV, older patients experienced fewer improvements in dizziness symptoms compared with younger people (2); residual dizziness (RD) after successful CRM is the major reason. The reported incidence of RD approach 30%–61%, and the duration of RD can range from a few weeks to several months (3–5). However, there were no clear risk factors for RD in middle-aged and older adults. The purpose of this study was to analyze risk factors of RD after successful treatment for BPPV in middle-aged and older adults.

## Patients and Methods

### Patients

Between March 2019 and July 2021, 181 patients with BPPV, after successful CRM treatment, were recruited at the neurotology unit. All patients had undergone routine nervous system and laboratory examinations. The diagnosis of BPPV was performed using the diagnostic criteria established by the Bárány Society in 2015 (6). The exclusion criteria for this study were: (1) multicanal, secondary, and superior canal BPPV; (2) a history of prior BPPV; (3) CRM treatment failure (more than 3 CRMs); (4) coexisting other vestibular disorders, such as vestibular neuritis and vestibular migraine; (5) central nervous system disorders; and (6) patients under the age of 45 years. All patients were screened by the treating physician for enrollment. The study was approved by the Institutional Review Board of Xijing Hospital, Air Force Medical University.

### Canalith Repositioning Maneuvers

Patients with posterior semicircular canal BPPV were treated by Epley's maneuver (7). Patients with geotropic lateral canal BPPV were treated by barbecue rotation maneuver (8). Patients with apogeotropic lateral canal BPPV were treated by the Gufoni maneuver (9). A Dix–Hallpike or Roll test maneuver was repeated about 60 min after the treatment and CRM was defined as “successful” if positional nystagmus was no longer detectable and the patient did not complain of vertigo. Otherwise, the first CRM was defined as “ineffective.” The patients of successful CRM treatment had undergone 3–7 days follow-up evaluation *via* interviews at the outpatient clinic after the first visit. During the follow-up, patients, again, underwent diagnostic positional tests (Dix-Hallpike or Roll test). We used videonystagmography to observe nystagmus (eliminating visual fixation). In case of negative test results (absence of positional vertigo and undetectable positional nystagmus), the CRM was defined as “successful.” Once successful treatment was confirmed, the occurrence of RD was subsequently recorded. Based on successful CRM treatment, we performed a structured interview procedure: (1) the first question, aimed to identify the persistent dizziness symptoms. We asked, “Do you still feel unsteadiness, lightheadedness, disorientation, fogginess, or drowsiness during the past days?”. In case the answer was “YES,” we will proceed to ask the next question; (2) the second question, aimed to verify the onset of RD. We asked “Is there a complete relief for symptoms after CRM. If the answer was “No,” the patient was considered to have RD.

### Clinical Variable Evaluations and Collection

All patients were divided into the middle-aged group (aged 45–59 years) and the older group (over 60 years). The clinical characteristics were recorded, including age, gender, numbers of maneuvers, involved canal, affected side, RD, and comorbidities (hypertension and diabetes). All patients needed to complete the Chinese version of the dizziness handicap inventory (DHI) questionnaire at follow-up (10). DHI score≤30 indicates normal to mild dizziness; DHI score>30, moderate to severe dizziness. The presence of perceived anxiety was evaluated with the Generalized Anxiety Disorder's 7-item scale (GAD-7) at follow-up. A score of ≤9 points indicates normal to mild anxiety; >9, moderate to severe anxiety (11).

### Statistical Analysis

Statistical analysis included Student's *t*-test for continuous variables, the chi-square test, and Fisher's exact test to compare proportions. Univariate logistic regression analysis was performed to identify the factors that were relatively associated with RD. Multivariate logistic regression analysis was performed with the relatively associated factors. Statistical analysis was done by using SPSS software (version 19, SPSS Inc., Chicago, IL, USA) with a statistical significance of *p* < 0.05.

## Result

In all, 181 patients with BPPV were included in our study. There were 101 (55.80%) middle-aged patients (69 female and 32 male), and 80 (44.20%) older patients (59 female and 21 male). The average ages of the two groups were 51.98 ± 4.29 years and 70.17 ± 7.29 years. Clinical characteristics of middle-aged and older-aged adults with BPPV are described in Table 1. The incidence of RD in the older group was significantly higher than that of the middle-aged group (*p* = 0.033). There was no difference in gender, numbers of maneuvers, involved canal, affected side, and comorbidities between the middle-aged group and older group (*p* > 0.05).

**Table 1:** Clinical characteristics of middle-aged and older adults with BPPV.

**Features**	**Middle-aged patients (45–59 years, *n* = 101)**	**Older patients (over 60 years, *n* = 80)**	***p*-value**
Age (years, mean±SD)	51.98 ± 4.29	70.17 ± 7.29	-
Gender (%)			0.425
Female	69 (68.31)	59 (73.75)	
Male	32 (31.69)	21 (26.25)	
Numbers of maneuvers (%)			0.069
1 Maneuver	78 (77.22)	52 (65.00)	
≥2 Maneuvers	23 (22.78)	28 (35.00)	
Involved canal (%)			0.973
Posterior	73 (72.27)	58 (72.50)	
Horizontal	28 (27.73)	22 (27.50)	
Affected side (%)			0.689
Right	50 (49.50)	42 (52.50)	
Left	51 (50.50)	38 (47.50)	
Residual dizziness (%)	42 (41.58)	46 (57.50)	0.033
DHI scale (%)			0.120
Normal to mild dizziness	67 (66.33)	44 (55.00)	
Moderate to severe dizziness	34 (33.67)	36 (45.00)	
GAD-7 scale (%)			0.050
Normal to mild anxiety	71 (70.30)	45 (56.25)	
Moderate to severe anxiety	30 (29.70)	35 (43.75)	
Hypertension (%)	29 (28.71)	33 (41.25)	0.078
Diabetes (%)	15 (14.85)	18 (22.50)	0.186

Among the 181 middle-aged and older patients, there were 88 (48.62%) with RD and 93 (51.38%) without RD. We performed both univariate and multivariable analyses to identify the possible factors associated with RD in middle-aged and older-aged patients. In Table 2, a comparison between patients with RD and without RD is shown. Age (*p* = 0.001), DHI scale (*p* = 0.016), GAD-7 scale (*p* = 0.010), and hypertension (*p* = 0.014) explained the statistical difference between the two groups. Results of the multivariate analysis are displayed in Table 3. It showed that age (odds ratio = 1.042, 95% CI: 1.012–1.074, *p* = 0.006), moderate to severe dizziness (odds ratio = 2.017, 95% CI: 1.053–3.865, *p* = 0.034), and moderate to severe anxiety (odds ratio = 2.228, 95% CI: 1.157–4.290, *p* = 0.017) were independently associated with RD.

**Table 2:** Univariate analysis of factors associated with residual dizziness in middle-aged and older adults with BPPV.

**Factors**	**Residual dizziness (*n* = 88)**	**No residual dizziness (*n* = 93)**	***p*-value**	**Odds ratio**	**95% CI**
Age (years, mean ± SD)	62.72 ± 10.47	57.46 ± 10.43	0.001	1.049	1.019–1.080
Gender (%)			0.291	1.413	0.743–2.688
Female	59 (67.04)	69 (74.19)			
Male	29 (32.96)	24 (25.81)			
Numbers of maneuvers (%)			0.085	1.773	0.920–3.418
1 Maneuver	58 (65.90)	72 (77.42)			
≥2 Maneuvers	30 (34.10)	21 (22.58)			
Involved canal (%)			0.058	1.888	0.973–3.662
Posterior	58 (65.90)	73 (78.49)			
Horizontal	30 (34.10)	20 (21.51)			
Affected side (%)			0.828	1.067	0.595–1.911
Right	44 (50.00)	48 (51.61)			
Left	44 (50.00)	45 (48.39)			
DHI scale (%)			0.016	2.120	1.152–3.898
Normal to mild dizziness	46 (52.27)	65 (69.89)			
Moderate to severe dizziness	42 (47.73)	28 (30.11)			
GAD-7 scale (%)			0.010	2.267	1.218–4.220
Normal to mild anxiety	48 (54.54)	68 (73.11)			
Moderate to severe anxiety	40 (45.46)	25 (26.89)			
Hypertension (%)			0.014	2.185	1.167–4.092
Yes	38 (43.18)	24 (25.80)			
No	50 (56.82)	69 (74.20)			
Diabetes (%)			0.713	1.152	0.542–2.452
Yes	17 (19.32)	16 (17.20)			
No	71 (80.68)	77 (82.80)			

**Table 3:** Multivariate analysis of factors associated with residual dizziness in middle-aged and older-aged patients.

**Factors**	**Odds ratio**	***p*-value**	**95% CI**
Age	1.042	0.006	1.012–1.074
Moderate to severe dizziness (DHI score>30)	2.017	0.034	1.053–3.865
Moderate to severe anxiety (GAD-7>9)	2.228	0.017	1.157–4.290
Hypertension	1.864	0.067	0.958–3.626

## Discussion

This study demonstrated that RD was prone to occur in older adults. Age, moderate to severe dizziness, and moderate to severe anxiety were independently associated with RD when further analyzing the factors associated with RD in middle-aged and older adults.

Age had a strong effect on the incidence of BPPV, with a dramatic increase after age 60 years old (1). This is expected because one of the key hallmarks of aging is the deterioration of the body systems, rendering the elderly more prone to osteoporosis, vitamin D deficiency, diabetes, hypertension, and hyperlipidemia. These factors also predisposed an individual to the development of BPPV (12, 13). Besides, although CRM was effective in BPPV management, many elderly patients still experienced postural unsteadiness, lightheadedness, disorientation, fogginess, or drowsiness at least 1 month after repositioning (2). These symptoms are referred to as “residual dizziness.” In our study, we found that more than half of elderly patients with BPPV, after successful CRM, experienced RD and the incidence of RD in the older group was significantly higher than that of the middle-aged group. Previous studies reported that the incidence of RD was 56%–63% in elderly patients (5, 14). Our finding is similar to these studies. The cause of RD is still under investigation and risk factors for RD need to be identified. Therefore, we further explore factors associated with RD in middle-aged and older adults with BPPV. The results indicated that age, moderate to severe dizziness, and moderate to severe anxiety were independently associated with RD. Due to the age-associated decline in the sensory system (vestibular, visual, and proprioceptive systems), the possible reason was that a longer time was needed for central adaptation after successful particle repositioning in middle-aged and older patients (15, 16). Besides, RD could result in a significant negative impact on middle-aged and older patients' daily function and quality of life. It is necessary for proper intervention. Current treatment modalities included medicine and vestibular rehabilitation. Betahistine was one of the most used drugs for the treatment of RD. Some studies reported that betahistine could effectively improve the symptom of patients with RD (17, 18). Besides, vestibular rehabilitation (VR) was also effective for the treatment of RD (19). The VR is a form of physical therapy using head and trunk movements and improves balance by developing vestibular system stimulation and central compensation (20). Also, VR is a simple, low-cost, and effective treatment method for improving balance, control, and quality of life in patients with BPPV, promoting visual stabilization with head movements, improving vestibular–visual interaction during head movement, and expanding static and dynamic posture stability (21).

The DHI scale is a survey that includes the functional, emotional, and physical effects of dizziness on an individual. It was already used to assess the RD after successful BPPV treatment (17). In our study, we found that patients with moderate to severe dizziness had a 2.017-fold higher risk of RD compared to those with normal to mild dizziness. This finding suggested that the DHI might represent a simple tool to predict the occurrence of RD. In addition, high anxiety score was also a risk factor for the occurrence of RD. Patients with moderate to severe anxiety had a 2.228-fold higher risk of RD compared to those with normal to mild anxiety. Some studies also reported that patients with RD had higher anxiety scores than patients with no RD (5, 18). This is consistent with the result obtained by us. It might be attributed to the adverse effect of anxiety in recovery from successful CRM for BPPV (22, 23). Therefore, GAD-7 scale scores could be used as indicators of adjuvant anti-anxiety medication use to relieve RD symptoms. Previous research also confirmed that anxiolytics reduce RD after successful CRM in patients with BPPV (22).

## Conclusion

In conclusion, older adults exhibited higher incidence of RD after successful treatment for BPPV. Age, moderate to severe dizziness, and moderate to severe anxiety were independent risk factors of RD in middle-aged and older adults.

## Data Availability Statement

The original contributions presented in the study are included in the article/supplementary material, further inquiries can be directed to the author/s.

## Ethics Statement

The studies involving human participants were reviewed and approved by Institutional Review Board of Xijing Hospital, Air Force Medical University. Written informed consent from the patients or patients legal guardian/next of kin was not required to participate in this study in accordance with the national legislation and the institutional requirements.

## Author Contributions

WF: study design, analysis of data, and manuscript drafting. YB and FH: study design and collection data. XA and YS: collection data. JH and XW: guiding the study. All authors contributed to the article and approved the submitted version.

## Funding

This study was supported by the National Natural Science Foundation of China (No. 82202788), Key R&D Program of Shaanxi Province (No. 2022SF-283), and the National Key R&D Program of China (No. 2018YFC2000300).

## Conflict of Interest

The authors declare that the research was conducted in the absence of any commercial or financial relationships that could be construed as a potential conflict of interest.

## Publisher's Note

All claims expressed in this article are solely those of the authors and do not necessarily represent those of their affiliated organizations, or those of the publisher, the editors and the reviewers. Any product that may be evaluated in this article, or claim that may be made by its manufacturer, is not guaranteed or endorsed by the publisher.
